# Supplementary material for: Mammalian gene expression variability is explained by underlying cell state
Source: Mol Syst Biol. 2020 Feb 11;16(2):e9146. doi: 10.15252/msb.20199146 (PMC7011657; doi:10.15252/msb.20199146)
Supplement: Supplementary file 1 — Appendix [file MSB-16-e9146-s001.pdf]

Appendix

Mammalian gene expression variability is explained by underlying cell state

Robert Foreman[1], Roy Wollman[1,2,3]

1) Institute for Quantitative and Computational Biosciences, University of California, Los Angeles and Program in Bioinformatics and Systems Biology, University of California, San Diego.
2) Departments of Integrative Biology and Physiology and Chemistry and Biochemistry, University of California UCLA.
3) Corresponding author: rwollman@ucla.edu

Table of contents.

Appendix Figure S1. – Page 2

Appendix Figure S2. – Page 3

Appendix Figure S3. – Page 4

Appendix Figure S4. – Page 5

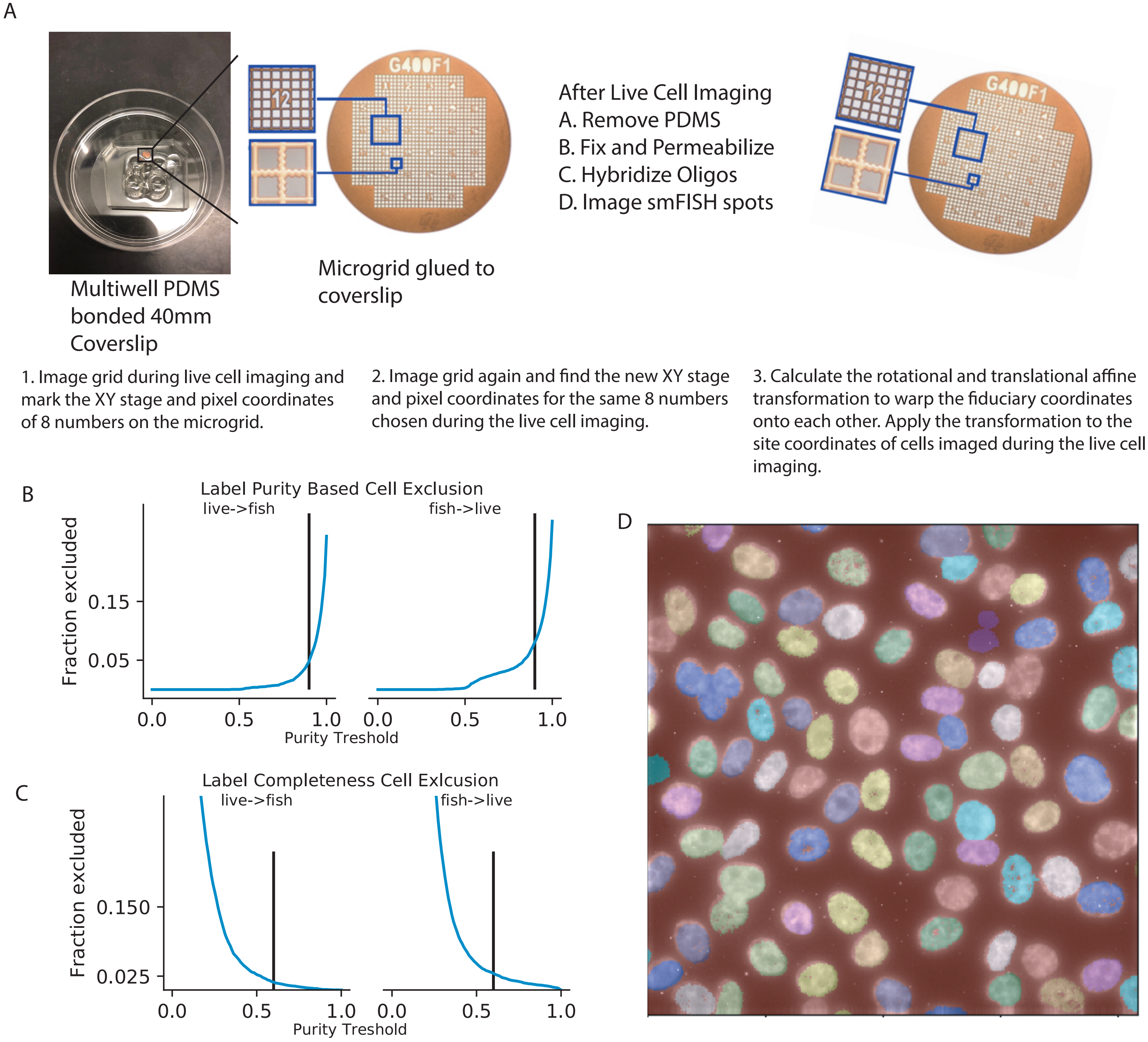

Appendix Figure S1 - Alignment of calcium images cells with smFISH imaging cells and quality control filtering of cells. (a) PDMS wells and the fiduciary grid attached to a 40mm coverslip (left). Example of orientation of grid during the live cell imaging (middle). Right panel shows that during the smFISH imaging the grid can be rotated, but coordinates of the fiduciary numbers are identifiable. (Below) description of the steps taken to align the fiduciary grids. (b) Purity of label is the fraction of pixels in the calcium nucleus labels that are the same as the nucleus labels from the FISH imaging (left), and vice-versa (right). Vertical lines represent thresholds used to discard cells which were not uniquely mapped between live calcium and fixed FISH imaging. (c) Label completeness is the fraction pixels that were non-zero in the paired segmentation and vice-versa (left, right). Vertical lines were the thresholds of completeness used in quality filtering of alignments.

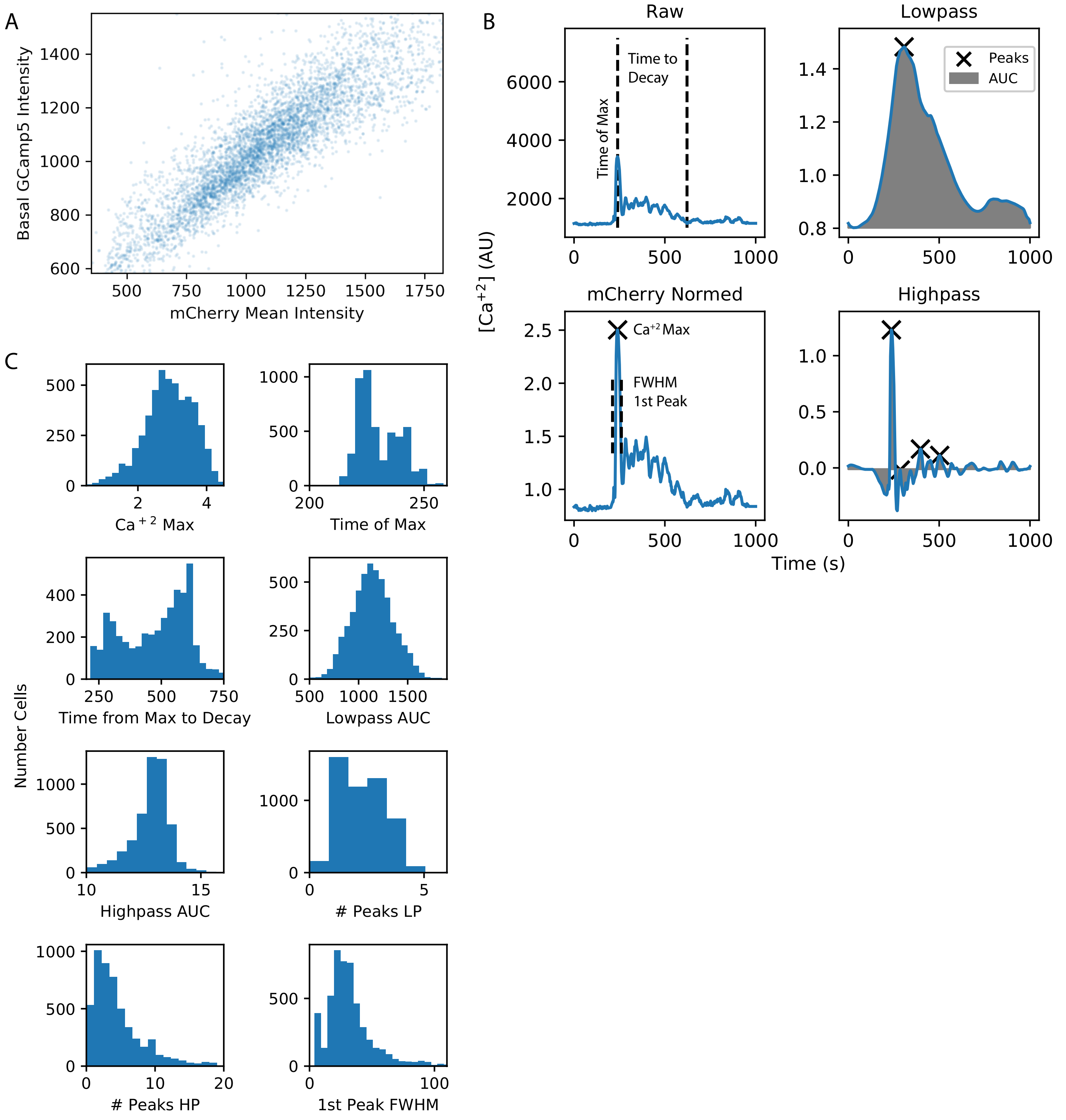

Appendix Figure S2 - Feature based representation of calcium trajectories. (a) Ca+2 is measured with GCaMP sensor fused with mCherry. The high correlation between basal GCaMP intensity and mCherry intensity indicates that mCherry intensity can be used to normalize sensor expression variability. (b) Top left shows raw GCaMP Ca+2 trajectories with the Time to Decay of 1st peak calcium feature, and the Time of Max feature for the example trajectory (dotted lines). Bottom left shows the mCherry normalized trajectory with the FWHM of 1st peak and the intensity value of Ca+2 Max feature for an example trajectory. Top right shows the lowpass filtered trajectory and the AUC (grey) as well as where the peaks in the lowpass are (x). Bottom right shows the same AUC (grey) and number of peaks (x) but for the high pass filtered trajectory. (c) Histograms of each calcium feature for all ~5000 cells are shown in each subpanel.

A

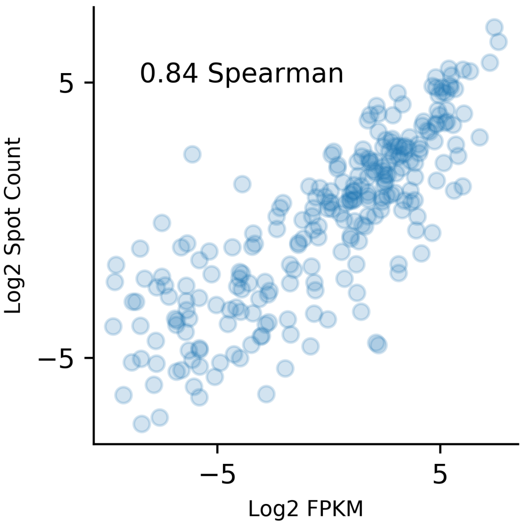

Appendix Figure S3 - Sequential hybridization smFISH is accurate measure of gene expression. (a) The scatter plot and correlation of RNA-Seq FPKM vs spot counts from the sequential smFISH measurements.

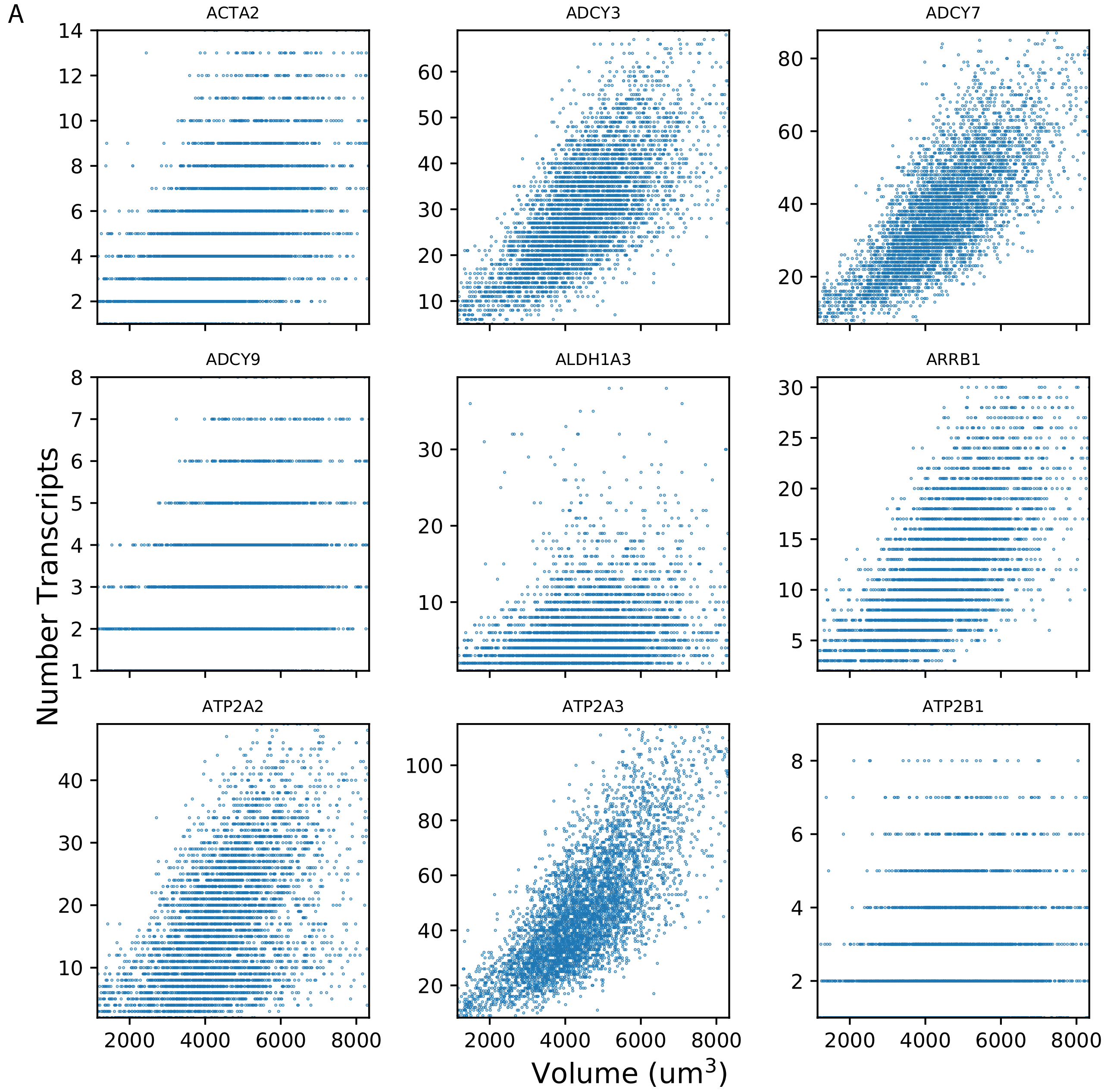

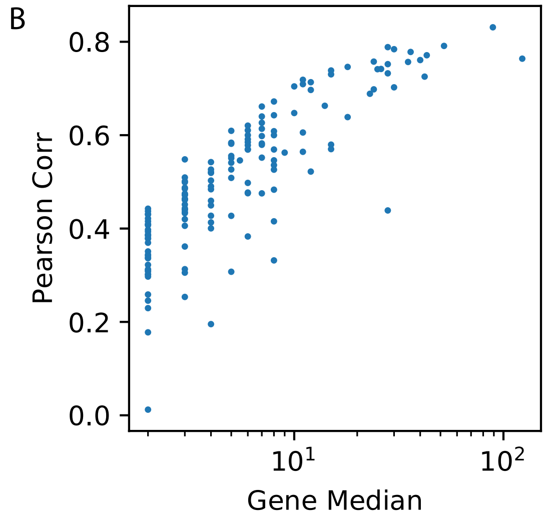

Appendix Figure S4 - The relationship between volume and gene expression counts for different genes. (a) Nine randomly selected genes are shown as a scatter plot of volume vs spot counts per cell. (b) The Pearson correlation of volume and gene expression as a function of the gene's median expression.
